# Socioeconomic-related health inequality in South Africa: evidence from General Household Surveys

**DOI:** 10.1186/1475-9276-10-48

**Published:** 2011-11-10

**Authors:** John E Ataguba, James Akazili, Di McIntyre

**Affiliations:** 1 Health Economics Unit, School of Public Health and Family Medicine, University of Cape Town, Observatory, 7925, South Africa; 2 Navrongo Health Research Centre, Ghana Health Service, P. O. Box 114, Navrongo, Ghana

**Keywords:** Socioeconomic health inequality, ill-health, South Africa

## Abstract

**Background:**

Inequalities in health have received considerable attention from health scientists and economists. In South Africa, inequalities exist in socio-economic status (SES) and in access to basic social services and are exacerbated by inequalities in health. While health systems, together with the wider social determinants of health, are relevant in seeking to improve health status and health inequalities, those that need good quality health care too seldom get it. Studies on the burden of ill-health in South Africa have shown consistently that, relative to the wealthy, the poor suffer more from more disease and violence. However, these studies are based on selected disease conditions and only consider a single point in time. Trend analyses have yet to be produced. This paper specifically investigates socio-economic related health inequality in South Africa and seeks to understand how the burden of self-reported *illness *and *disability *is distributed and whether this has changed since the early 2000s.

**Methods:**

Several rounds (2002, 2004, 2006, and 2008) of the South African General Household Surveys (GHS) data were used, with standardized and normalized self-reported illness and disability concentration indices to assess the distribution of illness and disability across socio-economic groups. Composite indices of socio-economic status were created using a set of common assets and household characteristics.

**Results:**

This study demonstrates the existence of socio-economic gradients in self-reported ill-health in South Africa. The burden of the major categories of ill-health and disability is greater among lower than higher socio-economic groups. Even non-communicable diseases, which are frequently seen as diseases of affluence, are increasingly being reported by lower socio-economic groups. For instance, the concentration index of flu (and diabetes) declined from about 0.17 (0.10) in 2002 to 0.05 (0.01) in 2008. These results have also been confirmed internationally.

**Conclusion:**

The current burden and distribution of ill-health indicates how critical it is for the South African health system to strive for access to and use of health services that is in line with need for such care. Concerted government efforts, within both the health sector and other social and economic sectors are therefore needed to address the significant health inequalities in South Africa.

## Introduction

Internationally, inequalities in health, especially with reference to the burden of ill-health on the poor, have received considerable attention among health scientists and economists [1]. Growing evidence points to the pervasiveness of such inequalities [2] but also to the fact that lower socio-economic groups suffer multiple deprivations [1,3]. This is also true across countries [4]. In South Africa, poverty, inequality in socio-economic status (SES) and inequality in access to basic social services between population groups, provinces, and socio-economic groups are typical and extensive [5,6] and these help to exacerbate inequalities in health. The poor face many predisposing factors that are recognised as social determinants of ill-health [2] but also they often cannot afford to seek care when ill. Further the response and coping strategies differ between the poor and the non-poor [7].

The health system has relevance to the social determinants of health and plays an important role in improving health status and addressing health inequalities [8]. However, the *inverse care law*, which suggests that the availability of good quality health care is inversely related to the need for it in the population it serves, seems to prevail in many countries [9-12]. A recent study in South Africa shows that the distribution of health service utilisation and of the benefits (measured in monetary terms) from using services is skewed in favour of the rich for most public facilities, especially hospitals, and across all private sector services [13]. These findings need to be compared to the distribution of ill-health across socio-economic groups.

Earlier studies on the burden of ill-health in South Africa (though using somewhat dated datasets), have consistently shown that, relative to the wealthy, the poor suffer more from most diseases and violence [14-18]. However, these studies are based on selected diseases and have considered only a single point in time. Trend analyses are yet to be conducted. In this regard, this paper investigates health inequality in South Africa from a bivariate perspective (health in relationship to socio-economic status) [19] and seeks to understand how the burden of *illness *and *disability *is distributed across socio-economic groups and whether this has changed since the early 2000s.

### Debates in inequality in health measurement

Inequalities in health may be defined as the variations in health status across individuals in the population [20]. This could be assessed univariately, bivariately or multivariately. Univariate analysis assesses inequality in the distribution of health in a population without reference to any other distribution. Bivariate analysis does so related to a second variable such as gender, region, or SES [19]. Multivariate analysis compares inequality in health simultaneously in relation to at least two other variables.

In the literature, inequality (absolute and relative)^1 ^may be analysed using six widely accepted measures - the range, the Gini coefficient, a pseudo-Gini coefficient, the index of dissimilarity, the slope index of inequality and the concentration index [21,22]. Other measures have been proposed and used by epidemiologists [see 19 for example]. For relative inequality however and in the case of the bivariate analysis involving SES, only the slope index of inequality and the concentration index have been shown to be consistent with ranking units across socio-economic groupings, sensitive to changes in population distribution across socio-economic groups and consistent with experience of health (or ill-health) across the distribution of SES [21,22].

In assessing socio-economic variations in health, usually national household survey datasets are used. With these, researchers face several challenges. One is the variation in reporting health and ill-health, especially for subjective measures [23,24]. Also, the sampling strategy used for household surveys does not directly take into account variations in the condition(s) under investigation across the population. Another challenge is that the experience of ill-health may vary across age-sex groups in the population. In these regards, particularly with respect to the latter, standardization is used to reduce the confounding effect of other variables on reported health (or ill-health). It is used to describe the distribution of health by socio-economic groups conditional on confounding demographic factors such as age and sex [22,25], using either direct or indirect procedures. Such standardization assumes that these confounding variables are correlated with health and the measure of socio-economic status. The direct form involves grouping individuals and obtaining a distribution of health (or ill-health) that would exist if all the groups had a similar age/sex structure. Indirect standardization attempts to correct the distribution of health by comparing it with that expected of the actual age/sex distribution [22,25]. Direct standardization however employs arbitrary groups that result in altering the value of the inequality measure depending on how many groups are selected [22]. Consequently it is indirect standardization that is used in this paper.

### Methodology for socio-economic related health inequality analysis

This paper uses concentration indices to assess the extent of socio-economic related inequality in the distribution of ill-health and disability across the population. For computational simplicity and convenience, the concentration index is defined as twice the covariance between the health (or ill-health) variable (*h*) and the rank of the SES measure (*r*) divided by the mean of the health variable (*μ*). The procedure reproduces the *unstandardized *concentration index.

To account for age-sex variations, an indirectly standardized concentration index is obtained by running a simple ordinary least squares (OLS) regression and obtaining an estimate *β *from

(1) $$2\sigma_{r}^{2}\left(h_{i}∕\mu \right)=\alpha +\beta r_{i}+\sum_{j}\gamma_{j}X_{ij}+\varepsilon_{i}$$

This estimate is interpreted as the indirectly standardized concentration index, where *x*_*ij *_are the confounding variables - age and sex, $\sigma_{r}^{2}$ is the variance of the rank (*r*) and *ε *is the stochastic error term [22,25]. The concentration index therefore measures the extent of inequalities in health (ill-health) that are systematically associated with socioeconomic status [21]. The indirectly standardized concentration index can also be obtained by subtracting the contributions of all standardizing variables from the unstandardized concentration index [26].

Another equivalent procedure for computing the indirectly standardized concentration index is discussed in O'Donnell *et al*. [25] and this procedure was used to standardized illness and disability used in generating the cumulative shares reported in this paper. Here, a prediction is obtained using the estimated coefficients ($\hat{\alpha }$ and ${\hat{\beta }}_{j}$.) from a simple

OLS regression as:

(2) $${ĥ}_{i}^{X}=\hat{\alpha }+\sum_{j}{\hat{\beta }}_{j}x_{ji}$$

where the variables remain as defined earlier.

The indirectly standardized health (or ill-health) variable ${ĥ}_{i}^{IS}$ is obtained using *h*_*i *_(individual health/ill-health value), ${ĥ}_{i}^{X}$ (estimated in Equation (2)), and $\bar{h}$ (the mean of the health or ill-health variable) simply as:

(3) $${ĥ}_{i}^{IS}=h_{i}-{ĥ}_{i}^{X}+\bar{h}$$

The theoretical value of the concentration index lies between -1 (i.e. when all the population's ill-health or disability is concentrated on the most disadvantaged person) and +1 (when all the population's ill-health or disability is concentrated on the least disadvantaged person) [21]. A value of zero indicates either that the population's ill-health is evenly concentrated along the distribution of socio-economic status or that on average, positive and negative effects cancel out across the distribution. Generally, a positive index signifies that the distribution of ill-health is higher among the richer SES groups while a negative index indicates the opposite. With dichotomous variables, however, and in large samples, the computed concentration index^2 ^will lie between *μ *-1 and 1- *μ *[27,28]. Therefore, to compare the standardized indices, Wagstaff [27] suggests further normalization of the standard index using (1- *μ)*. Recently Erreygers [29] noted that this is an *ad hoc *procedure that does not apply an axiomatic treatment in normalizing. He proposes some form of adjustment to the standard concentration index [30]. Following the observations in Erreygers [30], Wagstaff [28] defends the normalization of the standard concentration index noting that as opposed to other standard measures of inequality such as the standard concentration index, the normalized concentration index could fall or rise if everyone's health (or ill-health) rises by the same amount. Also the normalized concentration index is responsive to equiproportionate increases in health (or ill-health). The normalized index is however considered to be more responsive to equiproportionate increases in health (or ill-health) than to equal increments in health (or ill-health), especially when the mean of the variable remains unchanged. In summary, though there is little variation between the standard concentration index and the normalized index proposed by Wagstaff, the ordering of inequality has been shown to remain the same for both measures. However, Erreygers [29] notes that the normalization proposed in Wagstaff [27] "blow[s] up the levels of measured inequality for distributions with either high or low means" [29 p.523] while the measure proposed in Erreygers [30] obviates this situation.

For simplicity, as shown in Wagstaff [28], the Erreygers' [30] index (or correction of the concentration index) can be conveniently written as:

(4) $$E_{c}=\left(4\mu ∕b-a\right)\cdot C$$

where *C *= the standard concentration index, *μ *is the mean of the health variable, (*b *- *a*) is the range of the health variable. The Erreygers corrected concentration index (Ec) can also be obtained as a weighted formulation of Wagstaff s [27] normalized index. For simplicity, in the case of a binary variable, Wagstaff s normalized index (*Wc*) is given as:

(5) $$W_{c}=C∕\left(1-\mu \right)$$

Then the Erreygers index *E*_*c *_in (4), for the case of an indicator variable, can be written equivalently as:

(6) $$E_{c}=4W_{c}\left(\mu -\mu^{2}\right)$$

This means that the Erreyger's formulation can be obtained by scaling that obtained from Wagstaff s [27] normalization by the factor 4*μ*(1 - *μ*). As a result, unless otherwise specified, we present the results based on Wagstaff s [27] normalization.

### Data

Data were drawn from the 2002, 2004, 2006 and 2008 rounds of the nationally representative annual South African General Household Surveys (GHS) conducted by Statistics South Africa. The surveys employed a multi-stage stratified sampling design. Stratification is by provinces and then by urban and non-urban location within each province. These sampling design features were taken into account in all estimations. Relevant information extracted included the type of illness (flu/Acute Respiratory Tract infection (ART), diarrhoea, trauma, tuberculosis (TB), drug and substance abuse, depression, diabetes, high blood pressure (BP), HIV, and sexually transmitted disease (STD)) and disability (sight, hearing, speech, physical, intellectual, and emotional) that an individual suffers from as self-reported by the individual although, strictly, the presence of most of these illnesses can only be known through medical diagnosis. For disabilities, they have often been diagnosed or can be physically observed. The questionnaire presents the respondent with a list of conditions to respond to. The unit of our analysis is the individual. The recall period is one month for illnesses and six months as a minimum for the condition to be considered a disability.

### Constructing a measure of socio-economic status

It is important to note that the measure of inequality may be sensitive to the choice of socio-economic status measure [31]. In developing countries, and in datasets where household expenditure or consumption is available, it is preferred to household income as a measure of socio-economic status for inequality analysis. In this paper socio-economic status is however measured using composite indices of socio-economic status [see 32]. This is because our datasets do not contain reliable information on household income and expenditure. In order to ensure consistency these indices were constructed in each year using principal components analysis (PCA) on the same set of eleven variables (type of dwelling, roof, and wall material, access to safe drinking water, toilet, source of energy for lighting, and ownership of car, landline, cell phone, TV, and radio). This was then used to divide the population into quintiles. All analyses were performed in Stata^® ^version 11.

## Results

In what follows, we present the results from the analyses of various health conditions with accompanying *illness *or *disability *concentration indices. Note that all the results are based on age-sex standardized illnesses and disabilities. Tables 1 and 2 contain unstandardized and indirectly standardized means of illness and disability. The indirectly standardized means are very similar to the unstandardized means. In Table 3, the cumulative proportion of the burden of illness for 2008 is presented. The Table presents these shares and concentration indices for influenza (flu), diarrhoea, trauma, tuberculosis (TB), drug and substance abuse, depression, diabetes, high blood pressure (BP), human immunodeficiency virus (HIV) and sexually transmitted diseases (STDs). For these, only flu and diabetes have positive concentration indices signifying that the richer segment of the population reported these diseases more than did the poorer groups. The cumulative shares also show that for flu (and diabetes), the top 40% of the population bear over 43% (46%) of the burden compared to the bottom 40% of the population who bear about 37% (35%). For the rest of the conditions in Table 3 the burden on the bottom 40% of the population exceeds that on the top 40%. For TB, the bottom 40% of the population bear 65% of the burden compared to 17% for the top 40%.

**Table 1:** Unstandardized and indirectly standardized mean of Illnesses (2008)

	Q1(poorest)	Q2	Q3	Q4	Q5(richest)
Flu/ART	0.0638 (0.0637)	0.0772 (0.0764)	0.0726 (0.0724)	0.0870 (0.0852)	0.0846 (0.0813)
Diarrhoea	0.0097 (0.0092)	0.0082 (0.0078)	0.0078 (0.0073)	0.0036 (0.0036)	0.0032 (0.0029)
Trauma	0.0028 (0.0032)	0.0037 (0.0039)	0.0032 (0.0034)	0.0030 (0.0031)	0.0047 (0.0041)
TB	0.0123 (0.0123)	0.0100 (0.0098)	0.0064 (0.0062)	0.0053 (0.0047)	0.0017 (0.0002)
Drug abuse	0.0007 (0.0008)	0.0012 (0.0012)	0.0007 (0.0007)	0.0004 (0.0004)	0.0008 (0.0006)
Depression	0.0035 (0.0032)	0.0039 (0.0037)	0.0037 (0.0033)	0.0038 (0.0032)	0.0035 (0.0020)
Diabetes	0.0064 (0.0070)	0.0054 (0.0063)	0.0063 (0.0070)	0.0088 (0.0091)	0.0133 (0.0087)
High BP	0.0122 (0.0115)	0.0150 (0.0144)	0.0161 (0.0156)	0.0164 (0.0156)	0.0200 (0.0073)
HIV	0.0029 (0.0030)	0.0045 (0.0045)	0.0035 (0.0030)	0.0017 (0.0015)	0.0014 (0.0011)
STDs	0.0004 (0.0004)	0.0009 (0.0008)	0.0004 (0.0004)	0.0004 (0.0003)	0.0006 (0.0005)

Means of indirectly standardized variables are contained in parenthesis.

**Table 2:** Unstandardized and indirectly standardized mean of disabilities (2008)

	Q1(poorest)	Q2	Q3	Q4	Q5(richest)
Sight	0.0069 (0.0062)	0.0102 (0.0095)	0.0086 (0.0080)	0.0077 (0.0068)	0.0077 (0.0020)
Hearing	0.0061 (0.0058)	0.0055 (0.0052)	0.0049 (0.0048)	0.0040 (0.0038)	0.0053 (0.0025)
Speech	0.0027 (0.0025)	0.0032 (0.0029)	0.0020 (0.0018)	0.0018 (0.0015)	0.0022 (0.0015)
Physical	0.0143 (0.0146)	0.0131 (0.0133)	0.0090 (0.0091)	0.0110 (0.0105)	0.0088 (0.0035)
Intellectual	0.0062 (0.0058)	0.0065 (0.0059)	0.0056 (0.0049)	0.0054 (0.0044)	0.0048 (0.0037)
Emotional	0.0031 (0.0028)	0.0047 (0.0044)	0.0036 (0.0032)	0.0038 (0.0033)	0.0029 (0.0017)

Means of indirectly standardized variables are contained in parenthesis.

**Table 3:** Cumulative shares of illnesses in South Africa (2008)

**Quintiles of SES**	**Flu/ART**	**Diarrhoea**	**Trauma**	**TB**	**Drug abuse**	**Depressi on**	**Diabetes**	**High BP**	**HIV**	**STDs**
										
Poorest 20%	17.3%	30.7%	18.8%	37.1%	21.8%	21.2%	19.0%	17.8%	23.4%	17.0%
Poorest 40%	37.0%	55.1%	40.8%	64.9%	54.6%	44.0%	35.1%	39.0%	56.6%	51.0%
Poorest 60%	56.3%	78.9%	60.8%	83.3%	74.5%	65.4%	53.6%	62.8%	79.4%	67.7%
Poorest 80%	84.0%	93.0%	82.5%	100.0%	88.1%	90.3%	83.0%	91.8%	93.8%	83.7%
	
Standardized concentration index	0.0455** (0.0137)	-0.2248** (0.0306)	-0.0183 (0.0632)	-0.3344** (0.0311)	-0.1541 (0.1029)	-0.0662* (0.0375)	0.0123 (0.0439)	-0.0647** (0.0181)	-0.1976** (0.0421)	-0.1152 (0.1055)

Note: These are standardized for age-sex variations. Concentration indices are based on Wagstaff's normalization

Robust standard errors in parentheses

**, * significant at 1%, and 10% levels respectively

The distributions of cases of HIV and diarrhoea indicate that the bottom 40% of the population bears about 56% of the burden compared to 11% for the top 40%. Most of the concentration indices are statistically different from zero except for trauma, diabetes, drug abuse and STDs.

The disability results, based on the 2008 data presented in Table 4, consistently indicate that the poor suffer more disability compared to the richer SES groups. This burden is particularly high in physical, hearing, speech, and sight disabilities. The concentration indices in these cases are all negative and statistically different from zero at conventional levels of significance. About 53% of the physically disabled and also those with speech disability are concentrated in the poorest 40% of the population compared to about 30% among the top 40%. Emotional and intellectual disabilities are also more concentrated among the poor than the rich. About 46% of those reporting emotional disability are in the lower 40% of SES groups compared to about 34% in the top 40%.

**Table 4:** Cumulative shares of disabilities in South Africa (2008)

**Quintiles of *SES***	**Sight**	**Hearing**	**Speech**	**Physical**	**Intellectual**	**Emotional**
						
Poorest 20%	18.9%	26.6%	25.1%	28.5%	24.0%	18.5%
Poorest 40%	46.4%	49.3%	52.8%	53.1%	46.9%	45.5%
Poorest 60%	70.5%	70.7%	70.5%	70.4%	67.0%	66.3%
Poorest 80%	95.6% -	91.6% -	88.8% -	95.0% -	89.0%	92.0%
	
Standardized concentration index	0.11*** (0.029)	0.13*** (0.036)	0.11** (0.047)	0.15*** (0.024)	-0.08** (0.033)	-0.09** (0.036)

Note: These are standardized for age-sex variations. Concentration indices are based on

Wagstaff's normalization

Robust standard errors in parentheses

***, ** significant at 1%, and 5% levels respectively

**Figure 1:**
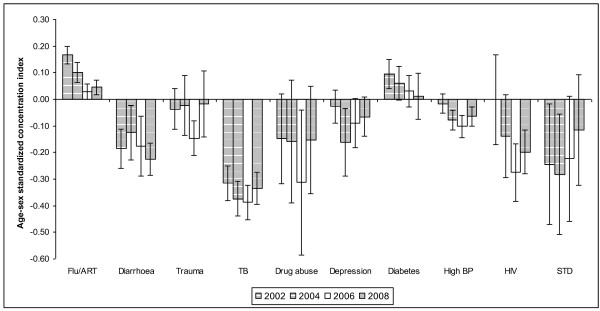
**Standardized illness concentration indices in South Africa (2002 - 2008) Note: The error bars represent 95% confidence intervals based on robust standard errors**.

**Figure 2:**
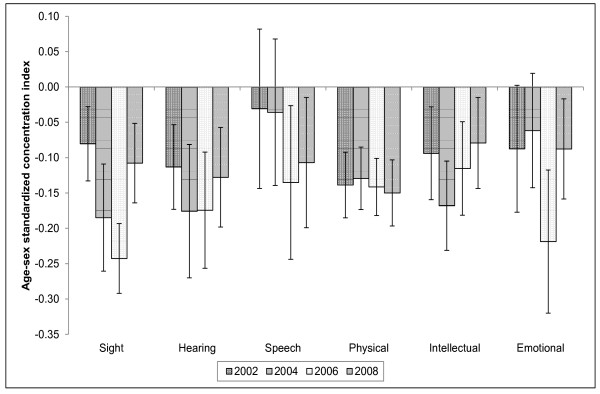
**Standardized disability concentration indices in South Africa (2002 - 2008) Note: The error bars represent 95% confidence intervals based on robust standard errors**.

The results presented in Tables 1, 2, 3 and 4 cover only the most recent dataset, that for 2008. To understand however if there are any changes in the patterns observed in 2008 from earlier periods, we present in Figures 1 and 2 the standardized *illness *and *disability *concentration indices based on data from 2002, 2004, 2006 and 2008. In Figure 1, the trend shows the same picture of the burden of different health conditions among different socio-economic groups as observed in Table 3. Again, only flu and diabetes have positive concentration indices signifying that the top 40% of the population bear more than 40% of the illness burden. Interestingly, for these two diseases, as indicated by the decline in concentration indices over time, they are not just concentrated among the rich but are increasingly being reported by the poor. For instance, the concentration index of flu (and diabetes) declined from about 0.17 (0.10) in 2002 to 0.05 (0.01) in 2008. In 2008, the concentration indices for flu and diabetes were close to zero. It is important also to note that the statistical significance of these indices varies as indicated by the 95% confidence interval bars. For HIV in 2002, the concentration index is approximately zero. This can be interpreted as statistical noise because of the relatively small number of data points and wide standard errors. Also to be noted is that the public roll-out of ART in South Africa, which contributed to an increased uptake of voluntary counselling and testing for HIV and hence awareness of one's HIV status, only commenced in 2003.

Unlike the cases of reported illness contained in Figure 1, the trends in concentration indices of disability conditions depicted in Figure 2 consistently indicate that there is a greater burden of all disability among the lower socio-economic groups compared to the richer groups. In most cases, these indices are statistically significant at conventional levels. Also in some cases the concentration indices have decreased in absolute value over the years. Some of these decreases may be indicative of disability becoming more broadly spread across the population rather than an improvement among the poor.

In Table 5 the cases of positive and negative concentration indices are shown to illustrate further the distinction between the diseases reported more by the poor and that by the rich. The concentration of TB is generally higher among the poor (negative indices). Reporting of flu is concentrated more among the non-poor (positive indices). These indices are statistically significant at conventional levels of significance.

**Table 5:** Trends in illness concentration indices in South Africa (2002 - 2008)

	Flu/ART		Tuberculosis (TB)	
		
	CI	Cumulative share	CI	Cumulative share
2002	0.1667*** (0.0168)	29% vs. 52%	-0.3156*** (0.0327)	57% vs. 16%
2004	0.1007*** (0.0195)	34% vs. 46%	-0.3746*** (0.0332)	66% vs. 16%
2006	0.0295** (0.0146)	39% vs. 40.3%	-0.3879*** (0.0335)	70% vs. 6%
2008	0.0455*** (0.0137)	37% vs. 44%	-0.3344*** (0.0311)	65% vs. 17%

Note: Robust standard errors in parenthesis. Concentration indices are based on Wagstaff's normalization.

Cumulative shares are represented as cumulative share of bottom 40% vs. top 40%.

***, ** significant at 1%, and 5% levels respectively

## Discussion

International evidence shows that lower socio-economic status is associated with a higher frequency of a variety of health problems [16,33-35]. A consistent negative association has been reported for birth weight, adult body height, prevalence of health complaints, prevalence of many chronic conditions, prevalence of disability, incidence of long-term work incapacity, perceived general health and adult mortality [35].

In South Africa also, a number of studies have documented higher frequency of key diseases among lower SES groups [see 14, 15, 16, 18, 36]. These studies, often based on a single disease, though informative, can only provide a partial picture of the overall burden of disease among different socio-economic groups in South Africa. This study attempts to provide evidence based on several health conditions to assess the socio-economic gradient of ill-health across a range of illnesses and disabilities. This will provide an indication of the relative 'need' for health care across different socio-economic groups.

This study found strong negative relationships between SES groupings and ill-health in most of the health conditions considered. This gradient is even more pronounced for disability. A few ill-health conditions, such as flu and diabetes, are concentrated among the richer rather than the poorer groups, but this is lessening over time. While the general burden of ill-health may be explained by social determinants such as socio-economic differences and life-style, in the case of disability the reasons for the observed negative gradients remain unclear [37].

In South Africa, as reported in this study and also by Myer *et al*. [16], there is a strong and persistent negative relationship between levels of SES and psychological distress, which is consistent with the international literature on the burden of mental diseases and distress [38-40]. Similarly tuberculosis is heavily concentrated among the poorest socio-economic group which is consistent with the results of Harling *et al*. [14] who note a high correlation between TB and cigarette smoking, alcohol consumption, low level of educational attainment, unemployment and poverty. The case of HIV/AIDS is similar to that reported by Cleary et al. [36] who found that in urban South Africa, HIV-positive individuals are concentrated amongst the poorer socio-economic quintiles. The distribution is also skewed more toward females than males. Diarrhoea, which is primarily a result of poor hygienic conditions, is more prevalent among the lower SES groups.

Although the General Household Survey does not have information on all diseases, it is important to note that those diseases that are included account for the majority of premature mortality, as measured by years of life lost (YLL). Bradshaw *et al*. [41] have estimated that HIV accounts for 39% of YLL, trauma (violence and road traffic accidents) for 10.5%, tuberculosis for 4.7% and diarrhoeal disease for 4.2% of YLL. These are the leading causes of premature mortality in South Africa, together constituting over 58% of YLL.

Of the diseases included in the General Household Survey and analysed here, HIV and other communicable diseases (often referred to as diseases of poverty) are more heavily concentrated among the poorest groups. Bradshaw *et al*.'s burden of disease study shows further that these two disease categories alone account for 62% of total YLL and 53% of Disability Adjusted Life Years (DALYs). Injuries (or trauma), which are also concentrated among lower socio-economic groups but to a lesser degree, account for just over 14% of YLL and DALYs. The final category of diseases, non-communicable diseases (NCDs), often referred to as diseases of affluence or of life-style, has more variable results. The only two NCDs included here are diabetes, which is more prevalent among higher than lower socio-economic groups although this is becoming less so over time, and hypertension, which is more concentrated among lower than higher income groups. Although this is a limited sample of NCDs (which account for 23% of YLL and 33% of DALYs), the results do suggest that these so-called diseases of lifestyle are becoming more evenly distributed across socio-economic groups in South Africa. This is again in line with international evidence [42-44]. Estimates indicate that by 2020, NCDs will be implicated in seven out of every ten deaths in developing countries [45,46].

The key limitation of this study is that the illnesses and disabilities considered are self-reported. For most of these conditions (e.g. HIV, STDs, TB, diabetes and hypertension), however, diagnosis at a health facility is the basis for the knowledge of the presence of the condition. This self-reporting, aided by facility-based diagnosis, may result in an underestimation among the poor because of their relatively low uptake and use of health services and therefore greater likelihood of suffering from undiagnosed illness. In some other cases, most especially the disabilities, but also for some illnesses such as diarrhoea, they are easily observable even without an official diagnosis at a health facility. It may be that only in a few instances, such as flu, is reporting based genuinely on a self-diagnosis. Two things however should be borne in mind when assessing the impact of this limitation. First, as noted previously, there is generally much lower self-reporting of illness among poorer groups. Sauerborn *et al*. [23] noted that, for these groups, "modifying illness perception (the phenomenon of ignoring disease)" is a key strategy for coping with the potential costs of illness. Many are simply too poor to be ill, in terms of either the loss of productive time if one acknowledges an illness or the costs associated with seeking health care [47]. Second, the poor use health services less than higher socio-economic groups. Lower socio-economic groups are, therefore, more likely than higher socio-economic groups to suffer from undiagnosed illness, particularly in terms of non-communicable diseases. Both these considerations suggest that, if anything, the socio-economic gradient reported here understates the true situation.

The results we have presented show the nature of the distribution of ill-health and disability that exist across socio-economic groups in South Africa. These results indicate a need for further research to probe the factors that affect and drive the observed distribution pattern of ill-health and disability in South Africa. Also relevant for future research is the need to understand why differences exist between the distribution of diabetes and high blood pressure, both of which are influenced by lifestyle behaviours.

## Conclusion

This study demonstrates that the burden of the major categories of ill-health and disability is greater among lower socio-economic groups in South Africa. Even non-communicable diseases, which are frequently seen as diseases of affluence, are increasingly being reported by lower socio-economic groups. It will be critical for government policies to tackle the underlying social determinants of ill health, including explicit attempts to address the conditions that predispose those who currently bear the greatest burden of ill-health to the risks of disease and disability [48]. It will take considerable time however for such policies to impact on health inequalities. Given the current burden of ill-health, and the inequality in its distribution, it is critical for the South African health system to strive for access to and use of health services that is in line with need for such care. At present, South Africa represents a classic example of the inverse care law; the lowest socio-economic groups bear the largest burden of ill-health (as illustrated in this paper) but have the lowest level of health service utilisation and derive the least benefits from service use [13]. Concerted government efforts, within both the health sector and other social and economic sectors, are required if we are to address the not insignificant health inequalities in South Africa.

## Competing interests

The authors declare that they have no competing interests.

## Authors' contributions

JEA was involved in the conception and design of the study, acquisition of data, data analysis, interpretation of data, drafting and revising the manuscript. JA and DM were involved in the conception and design of the study, data interpretation, drafting and revising the manuscript. All authors read and approved the final manuscript.

## Endnotes

1. A measure of relative inequality is not sensitive to a common scaling factor, say *α *>1. A measure of absolute inequality on the other hand is not sensitive to increasing the variable of interest by the same amount or quantity, say *β*. An example of a measure of absolute inequality is the absolute (or generalized) concentration index. In health data analysis, the majority of variables are cardinal or ordinal and often bounded within certain limits. Due to this, there is not such a clear distinction between absolute and relative measures of inequality [29].

2. In smaller samples, the corresponding bounds are (*μ *-1- (1/n)) and (1- *μ *+ (1/*n*)) where *n *= the sample size.
